# Book Reviews

**Published:** 1875-01-15

**Authors:** 


### Book Reviews.

CONTRIBUTIONS TO THE ANNALS OF MEDICAL PROGRESS, AND MEDICAL EDUCATION, IN THE UNITED STATES, BEFORE AND DURING THE WAR OF INDEPENDENCE. By Joseph M. Toner, M. D., Washington. Government Printing Office, 1874.

This is a volume of 118 pages, published by the Commissioner of Education, under the sanction of the Secretary of the Interior.

Although made up largely of short biographical sketches of the members of the medical profession, who practiced in the American colonies, prior to and during the War of Independence, yet it contains in addition a very valuable amount of information concerning the colonial legislation on medical subjects, and the state of medical education during that part of our history.

Indeed, it is a very valuable contribution to the history of the medical profession in America, and affords another illustration of the persevering industry and literary research of its distinguished author.

ON PRISON DISCIPLINE AND PENAL LEGISLATION, WITH SPECIAL REFERENCE TO THE STATE OF TENNESSEE. By J. Berrien Lindsly, M. D., D. D.

This is a pamphlet of 64 pages, printed in Nashville, and from the pen of one of the most gifted writers in our country. Although written with special reference to the treatment of criminals and the course of Penal Legislation in Tennessee, yet it contains an array of historical facts, both in regard to the general progress of penal legislation throughout the civilized world, and the local progress in this country, which renders it of much value to all who feel an interest in the progress of humanity.

ON ANCHYLOSIS. By Lewis A. Sayre, M. D., Prof. of Orthopœdic and Clinical Surgery, in Bellevue Hospital Medical College, New York. New York: D. Appleton & Co., 551 Broadway, 1874.

This is a neatly printed pamphlet of 20 pages, containing a paper on the general subject of Anchylosis, read to the New York Academy of Medicine, by Dr. Sayre, and originally published in the Transactions of the Academy, for October, 1874.

It may be read by all with interest and profit.

THE RELATIONS OF MEDICAL SOCIETIES TO PROGRESS IN SCIENCE. Inaugural Address of the President of the Medical Society of the County of Kings, New York, by Alex. J C. Skene, M. D., June 16, 1874, Brooklyn.

This is a pamphlet of 27 pages, devoted to a discussion of the three following propositions: "There are three avenues through which progress must come:

"1. Through the Medical Schools.

"2. Through Legislation by the People.

"3. Through the Profession, as Individuals and in Societies." While we could not agree with all the sentiments expressed by the author, we think his address may be read with interest.

AT a meeting of the students of the Chicago Medical College, January 7th, the following resolutions were unanimously passed:

WHEREAS, Recent severe sickness has rendered it necessary for our highly esteemed Professor of Organic Chemistry and Toxicology, WALTER S. HAINES, to seek temporarily, a milder climate; therefore

*Resolved*, That we, the students of the Chicago Medical College, deeply regret even the temporary loss of instruction from so skillful a teacher, and so kind a friend;

*Resolved*, That we hereby tender to him our sincere thanks for his recent masterly instruction, and zealous attention to our interests; and offer him our heartfelt sympathy and cordial hope for his speedy recovery and return to his post of honor and usefulness;

*Resolved*, That a copy of these resolutions be presented to the Professor, and also a copy to the CHICAGO MEDICAL EXAMINER, for publication.

(Signed) M. WARE, F. R. WEBB, J. R. KEWLEY, } Com.

---

THE ILLINOIS CHARITABLE EYE AND EAR INFIRMARY.—This institution now occupies its new building, corner of Adams and Peoria streets. The accommodations for the comfort and treatment of patients, it is believed, are not surpassed by those of any similar institution in the country. The upper stories are sub-divided into a large number of single sleeping rooms, instead of extensive wards; while the lower stories are provided with ample sitting-rooms, dining-hall, dispensary and clinic rooms. The building is heated with steam and provided with excellent means of ventilation. The edifice, 105 ft. long, by 47 ft. wide, consisting of a basement and four stories, with mansard roof, is an ornament to the portion of the city in which it is situated.

From the seventeenth annual report, we learn that 1,012 patients were treated gratuitously during the past year, making an aggregate of 10,620 that have received the benefits of this charity since its foundation in 1858. Regular clinical lectures on the diagnosis and treatment of diseases of the eye and ear, are given at the Infirmary during the winter as also the spring terms of our medical colleges. These lectures are open to medical students and practitioners from any portion of the country.

Patients are admitted for treatment, who bring certificates (signed by some respectable physician or county supervisor) either that they are absolutely unable to pay for their board or treatment, or that they can pay for their board alone.

The dispensary is open from 2 to 3 o'clock p. m., for out patients.

The following are the officers of the Infirmary: Trustees—E. W. Blatchford, President; B. W. Raymond, V. President; D. Goodwin, Jr., Secretary; H. W. King, J. T. Ryerson. Treasurer—E. B. McCagg. Consulting Surgeons—Prof. J. W. Freer; Prof. H. A. Johnson; Prof. E. Powell. Attending Ophthalmic Surgeons—E. L. Holmes, M. D.; F. C. Hotz, M. D. Attending Aural Surgeon—S. J. Jones, M. D. Microscopist—I. N. Danforth, M. D. Superintendent—G. Davenport. Matron—Mrs. Davenport.

